# Interaction between hydrogen and gallium vacancies in β-Ga_2_O_3_

**DOI:** 10.1038/s41598-018-28461-3

**Published:** 2018-07-04

**Authors:** Yidan Wei, Xingji Li, Jianqun Yang, Chaoming Liu, Jinyu Zhao, Yong Liu, Shangli Dong

**Affiliations:** School of Materials Science and Engineering, Harbin Institute of Technology, Harbin, 150001 China

## Abstract

In this paper, the revised Heyd-Scuseria-Ernzerhof screened hybrid functional (HSE06) is used to investigate the interaction between hydrogen with different concentrations and gallium vacancies in *β*-*Ga*_*2*_*O*_*3*_. The hydrogen can compensate a gallium vacancy by forming hydrogen-vacancy complex. A gallium vacancy can bind up to four hydrogen atoms, and formation energies decrease as the number of hydrogen atoms increases. Hydrogen prefers to bind with three coordinated oxygen. The bonding energy and annealing temperatures of complexes containing more than two hydrogen atoms are computed, and show relatively high stability. In addition, vacancy concentrations increase with the increasing vapor pressures. This paper can effectively explain the hydrogen impact in *β*-*Ga*_*2*_*O*_*3*_.

## Introduction

Hydrogen can be inevitable incorporated into *β*-*Ga*_*2*_*O*_*3*_ during its growth by means of metal organic chemical vapor deposition (*MOCVD*), or post growth processing steps which is needed to fabricate electronic and optoelectronic devices^1–3^. N-type conductivity in semiconductors has historically been attributed to native defects such as oxygen vacancies, but calculations and experiments have provided evidence that hydrogen can be responsible in the cases, which includes hydrogen in *α*- *Ga*_*2*_*O*_*3*_^4,5^. In *β*-*Ga*_*2*_*O*_*3*_, hydrogen is remarkably stable in gallium vacancy and interstitial location. Hydrogen is expected to be strongly bound at native defects such as gallium vacancies, and will significantly affect the characteristics of semiconductors^6,7^. It can be electrically active in semiconductors and behaves either as a donor or an acceptor, depending on its electrical activity and relative concentrations of other impurities and defects in the system^8,9^. Recently, gallium vacancies are found to have high concentrations, and can act as the role of acceptor or donor which can affect conductivity^10,11^. Therefore, it is interesting to investigate the interaction between hydrogen and gallium vacancies in *β*-*Ga*_*2*_*O*_*3*_. Previous work has mainly focused on the atomic structure, formation energy, and defect energy levels of gallium vacancy – hydrogen complex including only one hydrogen atom^8,12^. However, since the vacancy–hydrogen complex including more than one hydrogen atoms is easier to form than native vacancy and enormously affect vacancy levels in semiconductors, such as GaN and GaAs^13,14^. However, there has been no thoroughly report about interactions between hydrogen atoms and gallium vacancies in *β*-*Ga*_*2*_*O*_*3*_. Therefore, it is necessary to study the interaction between various numbers of hydrogen atoms and gallium vacancies, involving formation energies, bonding energies and concentrations of vacancy–hydrogen complex.

In this paper, the interactions between hydrogen atoms and gallium vacancies in *β*-*Ga*_*2*_*O*_*3*_ are characterized by the first principle calculation, including the formation energies for hydrogen-vacancy complexes containing up to four hydrogen atoms (*V*_*Ga*_*nH*, n = 1, 2, 3, or 4). From these results and previous ones for interstitial hydrogen, the reaction energy for the formula *V*_*Ga*_*nH* → *H*_*i*_ + *V*_*Ga*_(*n* − 1)*H* at various annealing temperatures, and vacancy concentrations under various vapor pressure from *pH*_*2*_ = 0.1 bar to *pH*_*2*_ ≈ 10^−21^ bar are also discussed.

## Computational Approach

The calculations in this study are based on the generalized Kohn–Sham theory with HSE06 screened hybrid functional and the projector augmented-wave method, as implemented in the VASP code^15–17^. The semicore [Ga] 3d^10^ and [O] 2p^6^ electrons were treated as core electrons. The cutoff energy for the plane wave basis was set to 400 eV. The structure was relaxed using conventional cell unless the force of each atom was not more than 1 meV/Å with a 3 × 5 × 5 Monkhorst-Pack Brillouin zone by GGA method.

Calculated properties of vacancy were carried out by using 2 × 2 × 2 point sampling, which has been proved reliable for analyzing different defects with the HSE functional method. To reduce finite-size effects, a periodic supercell of N = 120 atoms was used with computed equilibrium lattice constants, as listed in Table 1. In order to maximize the accuracy, the symmetry constrains were switched off and the spin polarization was taken into account.

**Table 1:** Calculated and experimental lattice parameters of *β*-*Ga*_*2*_*O*_*3*_.

Parameter	Our results calculated	Ref.^8^ HSE06	Ref.^21^ B3LYP	Ref.^22^ GGA + U	Refs^7,23,24^ Experiment
a(Å)	12.34	12.25	12.34	12.55	12.23 ± 0.02
b(Å)	3.07	3.05	3.035	3.08	3.04
c(Å)	5.86	5.84	5.799	5.89	5.80
β(deg)	103.67	103.9	103.7	103.67	103.7, 103.83
E_g_^d^(eV)	4.86	4.87	4.69	4.92	4.9

The formation energy of oxygen vacancy is calculated:

1 $${\rm{\Delta }}{E}_{V,q}^{f}({E}_{F})={E}^{defect}(q)-{E}^{perfect}(0)+{\mu }_{Ga}-{\mu }_{H}+q[{E}_{F}+{E}_{v}+{\rm{\Delta }}V],$$

where, *E*^*defect*^(*q*) is the total energy of a relaxed supercell containing vacancies with charge state q, *E*^*perfect*^(0) is the total energy of the pure host crystal. *E*_*F*_ is the Fermi level in the band gap with respect to the valence band maximum *E*_*v*_. It is necessary to adapt a potential-alignment with a correction term *ΔV* to correct the image charge. The energy is corrected with the method given by S. Lany and A. Zunger method^18,19^.

Equation (1) shows that defect formation energy depends on the chemical potential of the associated atomic species. The chemical potential is related to processing conditions, such as temperature and pressure, and this quantity is a variable in the formalism. The Ga atom out of the crystal lattice is placed in a reservoir with energy *μ*_*Ga*_, for which we make reference to the energy per Ga atom in the bulk. The chemical potential *μ*_*Ga*_ can vary to represent experimental conditions during the crystal growth or annealing, ranging from *O*-*rich* (*Ga*-*poor*) to *Ga*-*rich* (*O*-*poor*) conditions, which are bounds set by the formation enthalpy of *β*-*Ga*_*2*_*O*_*3*_:

2 $$2{{\rm{\mu }}}_{Ga}+3{{\rm{\mu }}}_{O}={\rm{\Delta }}{H}_{f}(G{a}_{2}{O}_{3})$$

In order to prevent formation of bulk Ga phases, O_2_ and H_2_, the chemical potentials are bounded as follows:

3 $${{\rm{\mu }}}_{Ga},{{\rm{\mu }}}_{O},{{\rm{\mu }}}_{H}\le 0$$

Similarly, the growth conditions reflect the range set by the formation enthalpy of each compound. To prevent formation of secondary H_2_O phases, the following condition is required:

4 $$2{{\rm{\mu }}}_{H}+{{\rm{\mu }}}_{O}\le {\rm{\Delta }}{H}_{f}[{{\rm{H}}}_{2}O]$$

Δ*H*_*f*_ (*Ga*_2_*O*_3_), and Δ*H*_*f*_[H_2_*O*] are formation enthalpies of Ga_2_O_3_, and H_2_O, respectively, and these can be calculated from the first principle. Combining Eqs (2) and (4) gives

5 $$2{{\rm{\mu }}}_{H}+\frac{1}{3}({\rm{\Delta }}{H}_{f}(G{a}_{2}{O}_{3})-2{{\rm{\mu }}}_{Ga})\le {\rm{\Delta }}{H}_{f}[{{\rm{H}}}_{2}O]$$

The inequalities in Eqs (3) and (5) allow us to describe the region of chemical potentials in the *μ*_*Ga*_-*μ*_*H*_ plane for which *Ga*-*O*-*H* is stable.

The charge state transition level ε(q/*q*′), describing the *E*_*F*_ at which the formation energy of two states with different charges is equal, is given by^20^

6 $${\rm{\varepsilon }}({\rm{q}}/q^{\prime} )=\frac{{\rm{\Delta }}{H}_{V,q^{\prime} }^{f}({E}_{F}=0)-{\rm{\Delta }}{H}_{V,q}^{f}({E}_{F}=0)}{q-q^{\prime} }$$

The formation energy is used to calculated defect concentrations. As entropy has an important effect on gibbs energy under high temperatures, it is needed to be considered. Therefore, in the strong dilution limit the concentration of oxygen vacancy is expressed as^4^

7 $$[{\rm{V}}]=\exp (\,-\frac{{{\rm{\Delta }}}_{f}{G}^{p,T}}{{k}_{B}T})$$

in which Δ_*f*_ *G*^*p*,*T*^ = Δ_*f*_*E* − TΔ_*f*_*S*^*p*,*T*^.

## Results and Discussion

### Bulk properties of β-Ga_2_O_3_

**Figure 1:**
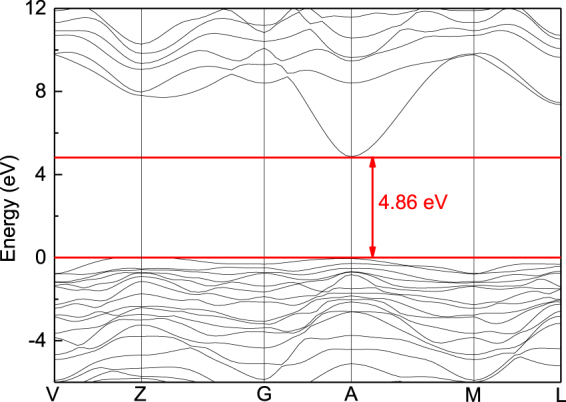
The band gap of *β*-*Ga*_*2*_*O*_*3*_.

According to the above computational approach, the lattice parameters of *β*-*Ga*_*2*_*O*_*3*_ are given in Table 1, which are in a good agreement with calculated and experimental results in the references^7,8,21–24^. The Hartree-Fock mixing parameter is set to 39%, which reproduces the band gap in Fig. 1.

### Formation energy of hydrogen-vacancy complexes

**Figure 2:**
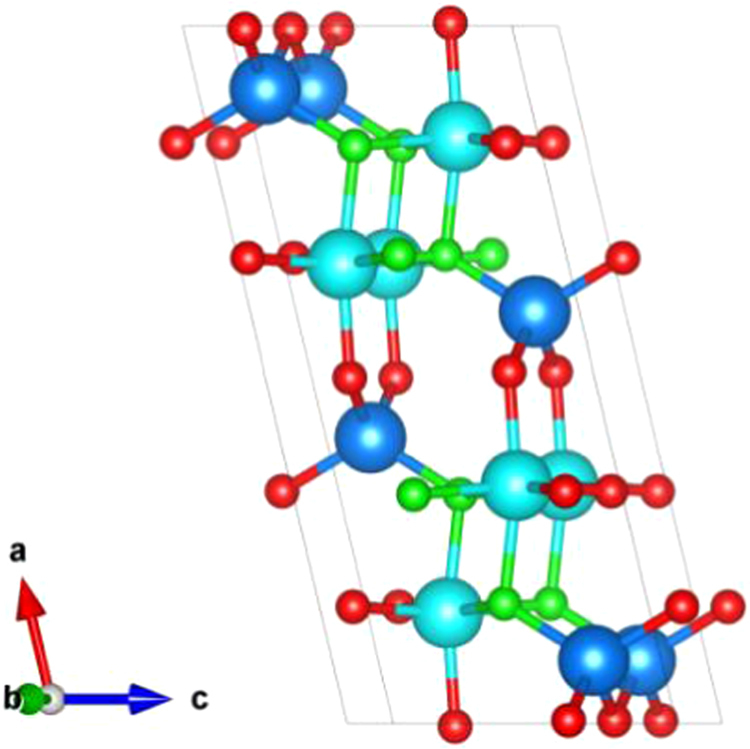
The structure of *β*-*Ga*_*2*_*O*_*3*_. (*Ga(I)* and *Ga(II)* are labeled as dark and light blue balls, *O(I)* or *O(II)* and *O(III)* are labeled as red and green balls.

The structure of *β*-*Ga*_*2*_*O*_*3*_ contains two inequivalent Ga sites and three inequivalent *O* sites, all of them located at 4i(x, 0, z) Wyckoff positions, as shown in Fig. 2. We label the sites as follows: *Ga(I)* is tetrahedrally coordinated and *Ga(II)* octahedrally coordinated. *O(I)* and *O(II)* are three-fold coordinated, while *O(III)* is four-fold coordinated. Moreover, their bonding environments are different, the two *V*_*Ga*_ sites lead to qualitatively similar band gap states. These states can accept up to three electrons for both inequivalent *V*_*Ga*_. Therefore, we investigate the gallium vacancy with up to three electrons. Moreover, complex vacancy containing more than four hydrogen atoms has no transition level in band gap which acts as shallow donor and cannot affect carrier recombination and electronic property. Therefore, we only consider the vacancy with no more than four hydrogen atoms.

**Figure 3:**
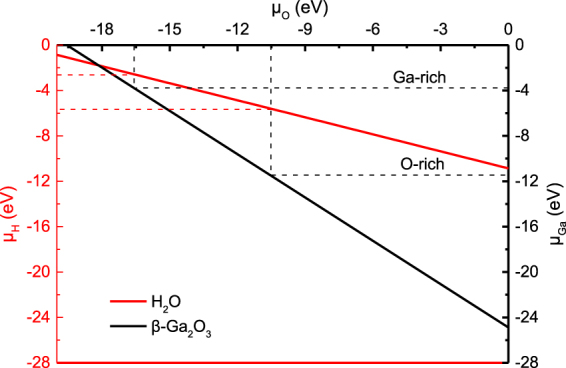
The region of chemical potentials. (Red and black lines are denoted to *H*_*2*_*O* and *β*-*Ga*_*2*_*O*_*3*_, respectively).

Considering Eqs (2)–(5), the region of chemical potentials in the *μ*_*Ga*_-*μ*_*H*_-*μ*_*O*_ plane for which *Ga*-*O*-*H* is shown in Fig. 3. From Fig. 3, it can be seen that potentials of hydrogen and gallium decrease when that of oxygen increases. Potentials can be obtained from intersections between potentials of *O*-*rich* or *Ga*-*rich* and their compound lines. Potentials of gallium, oxygen, and hydrogen are −3.78 eV, −16.58 eV, and −2.64 eV.

Previous density-functional-theory studies have examined the atomic structure, formation energy, and defect energy levels of *V*_*Ga*_*H* in *β*-*Ga*_*2*_*O*_*3*_^7^. In this paper, formation energy with different hydrogen numbers [*n* = *1*, *2*, *3*, *4*] is listed in Table 2 together with ref.^7^. According to Table 2, it can be seen that the formation energy decreases when the number of hydrogen increases. This is because the addictive hydrogen can effectively reduce dangling bonds resulting in lower the formation energy. And the number of electron the vacancy keeps decreasing, as the number of hydrogen increases. When it gets four hydrogen atoms, only the singly positive charged vacancy is stable within the band gap. There is not much available room for extra electrons, when hydrogen has dangling bonds. From Eq. (1), it can be acknowledge that the formation energy is closely connect with chemical potential reference. Gallium has many kinds of structures. Van de Walle uses chemical potential references coming from the α-phase of bulk Ga, while ones coming from the orthorhombic-phase of bulk Ga is used in this paper. The reason why choose this structure is that it has lower formation energy, which means a higher tendency to from. Meanwhile, the amount of exact exchange in the revised Heyd-Scuseria-Ernzerhof screened hybrid functional can affect the energy slightly. Therefore, reasons all above lead to differences of formation energy.

**Table 2:** Calculated charged vacancy formation energy obtained from Eq. (1) with *E*_*F*_ = 0 eV and Ga-rich. All values are in eV.

Charge	Our results HSE 06								Ref.^7^ HSE 06	
	V_Ga1_-H	V_Ga1_-2H	V_Ga1_-3H	V_Ga1_-4H	V_Ga2_-H	V_Ga2_-2H	V_Ga2_-3H	V_Ga2_-4H	V_Ga1_-H	V_Ga2_-H
1+	5.87	0.33	−4.04	−8.13	4.15	−0.80	−5.29	−9.88	6.47	5.85
0	6.76	2.23	−2.76		5.53	0.74	−3.61		7.63	6.64
1−	8.29	3.87			7.78	2.79			9.29	8.32
2−	10.50				10.30				11.97	10.86
3−	17.74				16.30					

**Figure 4:**
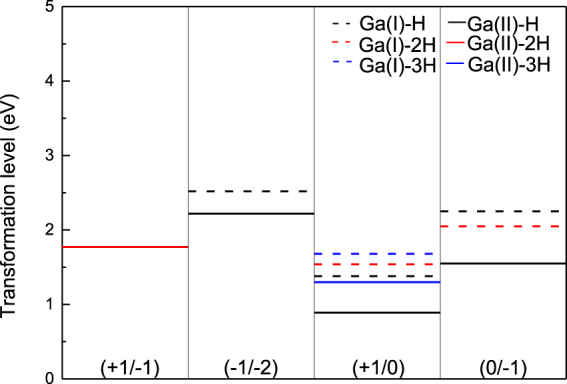
The transformation levels of charged *V*_*Ga*_*nH* vacancy (black, red, and blue lines are *V*_*Ga*_*H*, *V*_*Ga*_*2H*, and *V*_*Ga*_*3H*, respectively; the solid and dash lines are denoted as octahedral and tetrahedral gallium).

According to Eq. (6), the transformation levels with different number of hydrogen are shown in Fig. 4. The transformation levels of *V*_*Ga*_*nH* are all deep acceptors. But it can be seen vacancy related defect levels within the band gap is gradually removed as the number of hydrogen increases. For *Ga(II)*, *ε (*−*1*/−*2)* is removed when H increase to 2 H. And *ε (*+*1*/−*1)* changes from 1.818 to 1.773 which is also removed till the number of hydrogen increases to 3. All the transition levels are all removed when a gallium vacancy can bind up to four hydrogen atoms. For *Ga(I)* being contrary to *Ga(II)*, *ε (*+*1*/*0)* and *ε (0*/−*1)* change from 1.382 to 1.545 and 2.264 to 2.063, respectively. *ε (*−*1*/−*2)* is removed when *V*_*Ga*_*H* becomes *V*_*Ga*_*2H*. When the hydrogen number increases to 3, only *ε (*+*1*/*0)* is left. For *V*_*Ga*_*4H*, all defect levels are removed. According to Peter Déak’ results, it is found that the chosen epsilon can effectively affect transition levels^4^. However, in order to understand the interaction between hydrogen and gallium vacancy, and compare with C G Van deWalle, the static dielectric constant is used. The acceptor levels are effectively passive, when hydrogen atoms are added into the vacancy. However, contrary to C G Van deWalle^9^, the *ε (*+*1*/−*1)* replace *ε (+1*/*0)* and *ε (0*/−*1)* within the band gap for *Ga(II)*.

### Atomic structure of hydrogen-vacancy complexes

**Figure 5:**
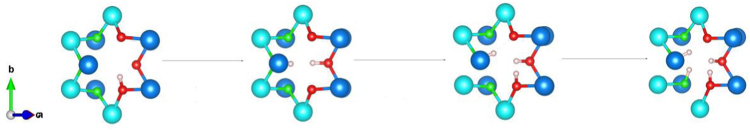
The stable system with different numbers of hydrogen when it is neutral. (*Ga(I)* and *Ga(II)* are labeled as dark and light blue balls, *O(I)* or *O(II)* and *O(III)* are labeled as red and green balls).

There are six oxygen atoms surrounding a vacancy site and hydrogen will likely passivate their dangling bonds. Half of coordinated oxygen atoms are three coordinated. We generated initial *V*_*Ga*_*nH* structures by placing hydrogen atoms in the vacancy site, and then relaxed these structures. After atomic structure relaxations, the corresponding stables with different numbers of hydrogen are shown in Fig. 5. By differentiating the connecting atom, hydrogen is more prior to bind to *O(I)* and *O(II)* than *O(III)*. When gallium vacancy generates, three coordinated oxygen is the relatively less stable than four coordinated ones. Therefore, hydrogen prefers to bind to *O(I)* and *O(II)*. Although hydrogen prefers to bind to three coordinated oxygen, there are only half of hydrogen atoms binding to them when hydrogen numbers increase up to four.

The distance between hydrogen and oxygen nearby is shown in Table 3. All distances are around 1 Å. And it can be seen distances increase when the bulk is with more electrons, meaning a relatively weaker bind between hydrogen and oxygen. Meanwhile, when more hydrogen is added, distances of earlier added hydrogen with oxygen in the same charged state increase. It is because hydrogen added later can effectively reduce the interaction between hydrogen added former and oxygen nearby. And when we compare octahedral gallium with tetrahedral ones, it is found tetrahedral ones are easier to bind with *O(I)* and *O(II)*.

**Table 3:** The distance between hydrogen and oxygen nearby.

Type	Charge	Octahedral		Tetrahedral	
		Oxygen			
*V*_*Ga*_ *nH*		Three-coordinate	Four-coordinate	Three-coordinate	Four-coordinate
*V*_*Ga*_ *H*	+1	0.9878		0.9860	
	0	0.9884		0.9884	
	−1	0.9898		0.9931	
	−2	0.9942		0.9995	
*V*_*Ga*_ *2H*	+1	0.9881	0.9881	0.9956	0.9856
	0	0.9878	0.9878	0.9924, 0.981	
	−1	0.9944	0.9944	0.9946, 0.9943	
*V*_*Ga*_ *3H*	+1	1.0130, 0.9887	0.9972	1.0073, 0.9827	0.9876
	0	1.0040, 0.9857	1.0236	1.0018, 0.9946	1.0083
*V*_*Ga*_ *4H*	+1	1.0251, 0.9844	1.0563, 0.9899	1.0119, 0.9926, 0.9799	0.9821

### Defect reactions of hydrogen-vacancy complexes

The actual defects present in a real system will depend on the processing, electron source, applied voltage, and temperature of that system. However, assuming an initial distribution of defects in different charged states and electron transfer between defects, we can combine the obtained information about the various defects with different charged states to make some predictions about which defect combinations are energetically more favorable.

Various reactions and their energies are presented in Table 4. These energies have been calculated as differences in total energies of pairs of individual defects and each pair has the same total charge state and number of atoms, where the binding energy is obtained from^25^

8 $${E}_{b}={E}_{f}({V}_{Ga}(n-N)H)+N{E}_{f}({H}_{i}^{+})-{E}_{f}({V}_{Ga}nH)$$

where ${E}_{f}({H}_{i}^{+})$ is the formation energy of interstitial H, *E*_*f*_ (*V*_*Ga*_(*n* − *N*)*H*), and *E*_*f*_ (*V*_*Ga*_*nH*) is the formation energy of the complex. When *n* − *N* = 0, *V*_*Ga*_ is the formation energy of the isolated vacancy.

**Table 4:** Defect reactions and associated energies.

No.	*V*_*Ga*_ *nH*	Reaction	E_b_ (eV)		Temperature (K)	
			Ga_1_	Ga_2_	Ga_1_	Ga_2_
1	*V*_*Ga*_ *H*	${V}_{Ga}H\to {{V}_{Ga}}^{-}+{{H}_{i}}^{+}$	3.58	4.56	1251	1564
2		${({V}_{Ga}H)}^{+}\to {V}_{Ga}+{{H}_{i}}^{+}$	2.82	3.26	1008	1149
3		${({V}_{Ga}H)}^{-}\to {{V}_{Ga}}^{2-}+{{H}_{i}}^{+}$	4.34	4.60	1494	1577
4		${({V}_{Ga}H)}^{2-}\to {{V}_{Ga}}^{3-}+{{H}_{i}}^{+}$	4.25	4.28	1465	1474
5	*V*_*Ga*_2*H*	${V}_{Ga}2H\to {({V}_{Ga}H)}^{-}+{{H}_{i}}^{+}$	3.12	4.10	1104	1417
6		${V}_{Ga}2H\to {{V}_{Ga}}^{2-}+2{{H}_{i}}^{+}$	7.46	8.70	2489	2885
7		${({V}_{Ga}2H)}^{+}\to {{V}_{Ga}}^{-}+2{{H}_{i}}^{+}$	7.07	7.95	2365	2646
8		${({V}_{Ga}2H)}^{+}\to {V}_{Ga}H+{{H}_{i}}^{+}$	3.49	3.39	1222	1190
9		${({V}_{Ga}2H)}^{-}\to {{V}_{Ga}}^{3-}+2{{H}_{i}}^{+}$	7.94	8.85	2642	2932
10		${({V}_{Ga}2H)}^{-}\to {({V}_{Ga}H)}^{2-}+{{H}_{i}}^{+}$	3.69	4.57	1286	1567
11		${V}_{Ga}2H\to {V}_{Ga}+{H}_{2}$	8.97	9.18	2971	3038
12		${({V}_{Ga}2H)}^{-}\to {{V}_{Ga}}^{-}+{H}_{2}$	7.33	7.13	2448	2384
13	*V*_*Ga*_3*H*	${V}_{Ga}3H\to {({V}_{Ga}2H)}^{-}+{{H}_{i}}^{+}$	3.69	3.46	1286	1213
14		${V}_{Ga}3H\to {({V}_{Ga}H)}^{2-}+2{{H}_{i}}^{+}$	7.38	8.03	2464	2671
15		${V}_{Ga}3H\to {{V}_{Ga}}^{3-}+3{{H}_{i}}^{+}$	11.63	12.31	3820	4037
16		${({V}_{Ga}3H)}^{+}\to {{V}_{Ga}}^{2-}+3{{H}_{i}}^{+}$	10.79	11.79	3552	3871
17		${({V}_{Ga}3H)}^{+}\to {({V}_{Ga}H)}^{-}+2{{H}_{i}}^{+}$	6.45	7.19	2167	2403
18		${({V}_{Ga}3H)}^{+}\to {V}_{Ga}2H+{{H}_{i}}^{+}$	3.33	3.09	1171	1095
19		${V}_{Ga}3H\to {{V}_{Ga}}^{-}+{{H}_{i}}^{+}+{H}_{2}$	11.02	10.59	3625	3488
20		${({V}_{Ga}3H)}^{+}\to {({V}_{Ga}H)}^{+}+{H}_{2}$	9.48	9.01	3134	2984
21	*V*_*Ga*_4*H*	${({V}_{Ga}4H)}^{+}\to {{V}_{Ga}}^{3-}+4{{H}_{i}}^{+}$	14.06	15.64	4595	5100
22		${({V}_{Ga}4H)}^{+}\to {({V}_{Ga}H)}^{2-}+3{{H}_{i}}^{+}$	9.81	11.36	3239	3734
23		${({V}_{Ga}4H)}^{+}\to {({V}_{Ga}2H)}^{-}+2{{H}_{i}}^{+}$	6.12	6.79	2062	2275
24		${({V}_{Ga}4H)}^{+}\to {V}_{Ga}3H+{{H}_{i}}^{+}$	2.43	3.33	884	1171
25		${({V}_{Ga}4H)}^{+}\to {{V}_{Ga}}^{-}+2{{H}_{i}}^{+}+{H}_{2}$	14.10	13.92	4608	4551
26		${({V}_{Ga}4H)}^{+}\to {({V}_{Ga}2H)}^{+}+{H}_{2}$	8.03	8.65	2671	2869

Positive energies indicate that a reaction in the direction of the arrow is energetically favorable. Note that we do not consider any reactions that include total energies with delocalized states. The energies presented in Table 4 also do not include the interaction between defects, which can be strong especially in close charged defect pairs. The formation energy of interstitial hydrogen atom and hydrogen is −2.94 eV and −0.43 eV, respectively. For *Ga(Ι)*, the formation energy of *E*_*f*_ *(V*_*Ga*_, *0)*, *E*_*f*_ *(V*_*Ga*_, −*1)*, *E*_*f*_ *(V*_*Ga*_, −*2)*, and *E*_*f*_ *(V*_*Ga*_, −*3)* is 11.63 eV, 13.28 eV, 15.57 eV, and 17.69 eV, respectively. And for *Ga(II)*, the formation energy of *E*_*f*_ *(V*_*Ga*_, *0)*, *E*_*f*_ *(V*_*Ga*_, −*1)*, *E*_*f*_ *(V*_*Ga*_, −*2)*, and *E*_*f*_ *(V*_*Ga*_, −*3)* is 10.35 eV, 13.03 eV, 15.32 eV, and 17.52 eV, respectively.

The activation energy *E*_*a*_ for dissociation of a defect complex can be estimated by the sum of binding and migration barrier of interstitial hydrogen^7^. We estimate the dissociation temperature based on an activated process with a hopping rate of the form *Γ* = *Γ*_*0*_*exp(*−*Ea*/*k*_*B*_*T)*^9^. A typical vibrational frequency *Γ*_0_ is estimated by ν = (2*E*_*a*_/*ml*^2^)^1/2^ ^26,27^. According to the formula, different charged states cannot enormously affect the vibrational frequency, therefore, neutral states are used to estimate vibrational frequency. Meanwhile, a distance around 3.1 Å is used as it is the distance of equivalent atom. As for Γ, it is not very sensitive and 1 min^−1^ is an appropriate value to evaluate temperature, which has been proved for many materials^12^. Therefore, we assume that dissociation starts occurring once the rate Γ reaches a value of 1 min^−1^, and Γ_0_ = 100 THz based on typical vibrational frequencies. Using this expression, we obtain an estimated dissociation temperature. Annealing temperatures of different reactions are also shown in Table 4.

As Table 4 shows, most of reactions are hard to diffuse at low temperature, due to its relatively high E_b_. Products composing of *V*_*Ga*_*nH*, or (*V*_*Ga*_*nH*)^−^ with ${H}_{i}^{+}$ have relatively lower annealing temperatures such as No. 1 and No. 5. However, (*V*_*Ga*_*nH*)^−^ is hard to anneal at this temperature. And we can see E_b_ and annealing temperatures increase with the number of ${H}_{i}^{+}$. Annealing temperatures of No. 11, No. 12, No. 20, and No. 26 are much higher than those of No. 6, No. 9, No. 17, and No. 24, which means the more hardness to form hydrogen gas than interstitial hydrogen. Annealing temperatures increase, when more hydrogen atoms are separated from complex vacancy. Moreover, interstitial H_2_ is harder to separate from complex vacancy than interstitial H.

### Defect concentrations of hydrogen-vacancy complexes

From the combination of the individual defect formation energy for HSE06, we have also calculated Schottky, and Frenkel energies for T = 0 K (all per created defect). The corresponding reaction equations for Frenkel and Schottky disorders are following.

Schottky equilibrium:

9 $${V}_{Ga}{H}_{H}^{X}\to {{V}_{Ga}}^{^{\prime} }+\frac{1}{2}{H}_{2}(gas)+{({V}_{Ga}H)}^{.}\,{{\rm{\Delta }}E}_{1}=7.11\,{\rm{eV}}\,{\rm{or}}\,6.37\,{\rm{eV}}$$

Frenkel disorder:

10 $${V}_{Ga}{H}_{H}^{X}\to {{V}_{Ga}}^{^{\prime} }+{H}_{i}^{+}\,{{\rm{\Delta }}E}_{2}=3.58\,{\rm{eV}}\,{\rm{or}}\,4.56\,{\rm{eV}}$$

For both two kinds of gallium vacancies, the Frenkel disorder energies are close and distinctly lower than the Schottky energies. It seems that Frenkel disorder is dominant.

Next, vacancy concentrations under different temperature and pressure are considered. The chemical potentials used for the calculation of the formation energy were constrained to lie within the stability field of *β*-*Ga*_*2*_*O*_*3*_ in Fig. 2. However, these values are only valid for T = 0 K. As we want to extend our analysis of the defect properties of gallium oxide to the case of T > 0 K, we also have to include the temperature dependence of the chemical potentials in our phase stability considerations. First, we take the T = 0 K total energy of the hydrogen molecule *E*_*tot*_*(H*_*2*_*)* to be approximately the enthalpy under reference conditions, i.e., temperature T_0_ = 298.15 K and pressure P_0_ = 1 bar. The remaining temperature and pressure dependence are taken from thermochemical tables,

When *β*-*Ga*_*2*_*O*_*3*_ is manufactured, the system locates in the Ga-rich region. And the hydrogen potential is expressed as^28^

11 $${\mu }_{H}(T,p)=\frac{1}{2}{E}_{tot}[{H}_{2}]+{\rm{\Delta }}{\mu }_{H}(T,{p}_{0})+\frac{1}{2}{k}_{B}\,{\rm{Tln}}(\frac{p}{{p}^{0}})$$

where, *E*_*tot*_[*H*_2_] is the total energies of hydrogen and Δ*μ*_*H*_(*T*, *P*_0_) is the change in chemical potential from the reference temperature T_0_ to temperature T > T_0_. According to the limiting phases, we also calculate *μ*_*Ga*_, in order to get a reliable and coherent phase stability range for the different temperatures. In this way, we can compute defect concentrations depending on partial pressures *pH*_*2*_ and compare our results directly to experimental data.

Δ*μ*_*H*_(*T*, *p*_0_) is the potential at certain temperature which is expressed as^29^:

12 $${\rm{\Delta }}{\mu }_{H}(T,{p}_{H})=\frac{1}{2}\{[{H}_{0}+{\rm{\Delta }}H(T)]-T[{S}_{0}+{\rm{\Delta }}S(T)]\}$$

where, Δ*H*(*T*) = *C*_*p*_(*T* − *T*_0_) and Δ*S*(*T*) = *C*_*p*_*ln*(*T*/*T*_0_) are the enthalpy and entropy changes, and H_0_ = 8.7 kJ mol^−1^ and entropy S_0_ = 205 J mol^−1^ K^−1^. And *C*_*p*_ is the constant-pressure heat capacity, equating to 29.4 J/(mol · K^−1^)^30^. We also chose the entropy of gallium vacancy for calculating the gibbs energy in Eq. 4, as it is very dilute in *β*-*Ga*_*2*_*O*_*3*_. And the entropy is −1.61 eV by the research of T. Zacherle^28^.

**Figure 6:**
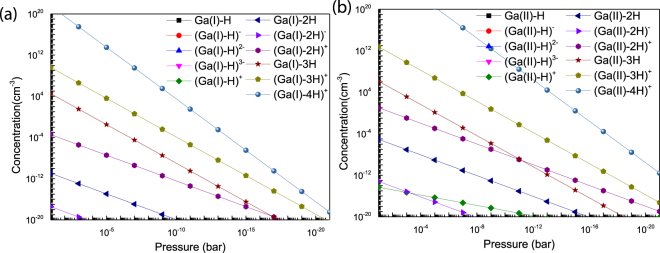
The defect concentration of complex vacancy against the hydrogen partial pressure with *T* = 1273 *K* and Ga-rich ((a) *Ga(I)*, (b) *Ga(II)*).

In Fig. 6, we display the defect concentration of complex vacancy against the hydrogen partial pressure for T = 1273 K. The calculated stability range is from *pH*_*2*_ = 0.1 bar to *pH*_*2*_ ≈ 10^−21^ bar. At high pressure, it is found that the concentration of positive charged *V*_*Ga*_*nH* is much higher than other charged states. All negative charged vacancies have much lower concentrations. Combining with the annealing temperature, the positive charged vacancies cannot be a major factor affecting the characteristic of devices, due lower concentrations and annealing temperatures. With the pressure decreasing, all vacancy concentrations change a lot and decrease to less than 1 cm^−3^. And concentrations of tetrahedral gallium are larger than octahedral ones.

## Conclusion

The stability of hydrogen - gallium complex vacancy in *β*-*Ga*_*2*_*O*_*3*_ is systematically discussed. It is found that gallium vacancy can bind up to four hydrogen atoms and the formation energy decreases and transformation levels are gradually disappeared with the hydrogen number increases. Moreover, V_Ga_3H and V_Ga_4H are predicted to be unstable in n-type *β*-*Ga*_*2*_*O*_*3*_, precluding complete passivation of gallium vacancies in n-type material. Hydrogen can either compensate a vacancy by donating an electron to a vacancy acceptor level, or passive the vacancy by forming a hydrogen-vacancy complex. The added electron can reduce the bond interaction between hydrogen and oxygen nearby. Hydrogen atoms prefer to bind with three coordinated oxygen atoms, then begin to bind with four coordinated oxygen when the number of hydrogen is more than one. By calculating bind energies, it is found that the complex vacancy with more than two hydrogen atoms is stable, which also has relatively high annealing temperatures. With more hydrogen atoms are separated from complex vacancy, annealing temperatures increase. Compared with interstitial hydrogen atom, interstitial H_2_ is harder to separate from complex vacancy. All vacancy concentrations decrease with the pressure decreasing. The vacancy filled with more hydrogen atoms has higher concentration. The gallium vacancy containing four hydrogen atoms has largest concentration among all kinds of vacancies. This paper can effectively explain the hydrogen impact in *β*-*Ga*_*2*_*O*_*3*_.
